# How the Rise of Problematic Pornography Consumption and the COVID-19 Pandemic Has Led to a Decrease in Physical Sexual Interactions and Relationships and an Increase in Addictive Behaviors and Cluster B Personality Traits: A Meta-Analysis

**DOI:** 10.7759/cureus.40539

**Published:** 2023-06-16

**Authors:** Ricardo Irizarry, Haley Gallaher, Steven Samuel, Jason Soares, Julia Villela

**Affiliations:** 1 Psychiatry, Tropical Texas Behavioral Health, McAllen, USA; 2 Medical School, Saint James School of Medicine, The Quarter, AIA

**Keywords:** problematic pornography consumption, cluster b personality traits, addictive disorders, covid-19, pornography consumption

## Abstract

On January 13, 2018, an alert was sent to Hawaii's people that a missile was heading toward them. People were in a state of alarm for 30 minutes before the government sent out a false alarm statement. Fifteen minutes after the message that told the people of Hawaii that they were not in danger went out, Pornhub's views spiked by 48%. On March 11, 2020, coronavirus disease 2019 (COVID-19) was designated a pandemic. By March 25, 2020, Pornhub's views had spiked to over 24%.

We took the research available on problematic pornography consumption, also referred to as internet sex addiction, pornography addiction, and cybersex addiction, and compared that to the rise of pornography use since the year 2000 and how the COVID-19 pandemic impacted pornography use and the effects it had on sexual and social relations. We also wanted to see if there is any association between pornography consumption and other addictive disorders and cluster B personality traits. There is currently no Diagnostic and Statistical Manual of Mental Disorders, Fifth Edition (DSM-5) diagnosis for pornography addiction. We want to see if the data we gather can aid in identifying whether problematic pornography use has a place alongside other addictive disorders in the DSM-5. We hypothesize that inappropriate pornography consumption has increased since 2000, only to increase further during the pandemic. The null hypothesis, Ho, states there has been no change in the consumption of pornography since the 2000s. The alternative theory, Ha, says that the proportion of people who use pornography has increased over the past 23 years. As for other addictive disorders and cluster B personality traits, we hypothesize the research will show that greater than 50% of people exhibiting problematic pornography consumption will also have an additional addictive disorder and a cluster B personality trait. Our results support our hypothesis that during the COVID-19 pandemic, pornography consumption increased beyond the baseline. The results did not support our prediction of a significant association between other addictive disorders and cluster B personality traits with pornography consumption*.*

## Introduction and background

The objective of this meta-analysis is to compare how the rise of pornography use and the coronavirus disease 2019 (COVID-19) pandemic has led to a decrease in sexual and personal connections and relationships while also correlating pornography use with other addictive behaviors, including cluster B personality traits. PubMed, Google Scholar, EBSCO Medline, JAMA, and ScienceDirect were used to establish and review existing literature on these topics.

The increase in pornography consumption has been a widely discussed topic in recent years regarding concerns about its potential effects on relationships and society [1]. This paper will explore the reasons behind the rise in pornography consumption and explore possible implications. One of the primary reasons for the increase in pornography consumption is the widespread availability of internet pornography [2,3]. Increased internet use allows pornography to be easily accessible to anyone with an internet connection [4]. The rise in porn consumption may also be attributed to societal attitudes toward sex. In recent years there has been a shift toward a more open and accepting attitude toward sexuality and sexual activity, which has made it more socially acceptable to view pornography. The destigmatization of sexual activities and sexuality has likely contributed to the increase in porn consumption [5].

The potential effects of the increase in pornography composition are varied and complex; we attempt to address the psychological aspect in this paper. Some studies we found in our research suggest that pornography can harm personality and relationships, leading to problems such as addictions, distorted views of sexuality, and decreased satisfaction in sexual relationships [1]. However, other studies have suggested that pornography can positively impact some individuals, such as providing a means of sexual education and increasing sexual pleasure [6].

Over 2.5 million people visit pornography sites every 60 seconds [7]. So what makes pornography consumption problematic? A Problematic Pornography Consumption Scale (PPCS) of 18 questions was made to distinguish between problematic and non-problematic pornography consumption. It is answered on a scale from one to seven; the higher the number, the more likely the individual will have problematic pornography use. The cut-off used for this scale is 76 [8]. Although not all articles in our paper used the PPCS, it is vital to address and define it here. It is also of note that PPCS is not used to diagnose addiction; in fact, there is no Diagnostic and Statistical Manual of Mental Disorders, Fifth Edition (DSM-5) diagnosis for pornography addiction or problematic pornography consumption. In this meta-analysis, we will discuss the avenues of pornography consumption and its effects, which can help discern if pornography consumption should have a place amongst other addictive disorders.

We will first cover the general trend of the rise of pornography consumption since the early 2000s and how the accessibility of pornography has affected the millennial and younger generations in terms of physical sexual connections, family life, and individual well-being. This cultural change includes the explosion of social media and its usage during this time [4]. Social media has reduced the barriers to viewing pornography. For example, some social media sites, such as Twitter, do not regulate erotic materials, making pornographic posts available to anyone [9]. Following is the debate over the impact that COVID-19 has had on sexual and personal relationships in younger generations. This analysis will encompass how the pandemic has impacted pornography usage and how social isolation fits into the change in sexual and intimate relations. Finally, we will discuss problematic pornography consumption and its link to other addictive disorders and cluster B personality traits.

Learning outcomes addressed in this paper will include how to define and recognize problematic pornography consumption, how the increase in accessibility to erotic content can influence undesirable behaviors, what cluster B personality traits are and how they correlate with addictive personality behaviors, and the associations between problematic pornography usage along with other addictive disorders and cluster B personality traits.

## Review

**Figure 1:**
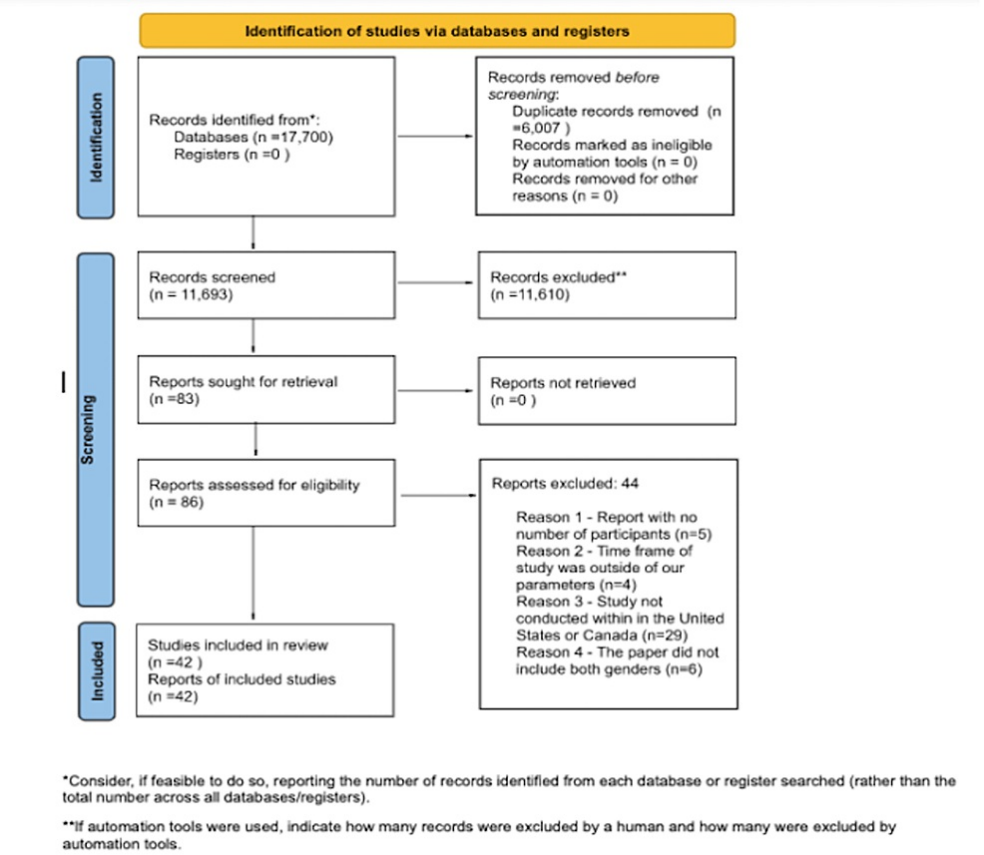
Preferred Reporting Items for Systematic Reviews and Meta-Analyses (PRISMA) The PRIMSA flow diagram maps out the number of records identified, included and excluded, and the reasons for exclusions. This template is from http://prisma-statement.org/prismastatement/flowdiagram.aspx.

This meta-analysis was performed congruently with Preferred Reporting Items for Systematic Reviews and Meta-Analysis (PRISMA) as shown in Figure 1 below. Our analysis follows a qualitative approach based on our results analysis of the articles used for this paper [10]. The inclusion criteria for all sections of this analysis include papers published after 1999, papers written in English, and papers inclusive of all genders. The exclusion criteria encompass studies performed outside the United States and studies performed specifically on a single group of individuals. To our knowledge, this is the first meta-analysis on this topic.

Throughout our study selection, 17,700 articles were found on the databases PubMed, Google Scholar, EBSCO, JAMA, and ScienceDirect. After removing duplicates, abstract screenings, and complete text screenings, we had 42 remaining articles to use in this paper. This exclusion was done by all authors independently. The same system was used to determine the papers for each section of this meta-analysis.

Data extraction

Part A: Rise in Pornography Viewing Since the Early 2000s and Effect on Masturbation vs. Partnered Sexual Acts

**Figure 2:**
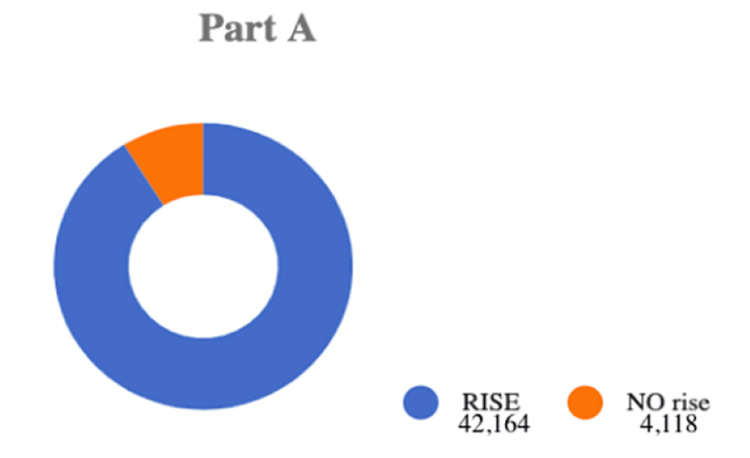
Illustration of the data showing the number of participants that showed an overall rise in pornography consumption. Showing, in graph form, the rise of pornography consumption since the year 2000.

All papers in this section of our analysis include participants who engage in solo sexual acts versus engaging in partnered sexual acts. The number of participants was calculated for each paper. The majority of participant ages range from 18 to 50 years old. All studies also include both men and women of any sexual preference. We gathered data using the total number of participants in each study. We used that to calculate whether there was an association between a rise in pornography consumption and an increase in solo masturbation versus engaging in sexual acts with another partner. We searched for articles that described the changes in pornography views since 2000 and how this changed the frequency of solo masturbation compared to partnered sexual acts. To conduct this search, we first looked at pornography consumption since 2000, then adjusted our search to pornography consumption's increased effect on masturbation as shown in Table 1. Of the papers found, 42 met our inclusion and exclusion criteria yielding a total of Nt = 57,173 participants. Of the total papers in part A, participants were N1 = 46,282. Of these participants, N2 = 42,164 had an increase in pornography consumption, and it was additionally concluded that viewing pornography contributed to increased solo masturbation. However, some papers showed no change, decreased pornography consumption, or evidence of more engagement with a partner (reduced solo masturbation), N3 = 4,118. In this category of participants, viewing pornography did not affect partnered sexual relations. By dividing N2 by N1 and multiplying that by 100, we got a result of a 91.10% overall increase in pornography consumption from the year 2000. We also calculated the pooled proportion of the data for part A which included all the papers that fit the criteria for an increase in pornography consumption. The data shows a rise in pornography consumption for part A. A pooled proportion test was done to determine the percentage of increase, which yielded results showing us a value of 0.911. This further supports the 91.10% overall increase in pornography consumption. Additionally, we theorize that participants who consume pornography are more likely to perform solo sexual acts instead of partnered sexual acts. Figure 2 illustrates the difference in the number of papers that depict a rise in pornography consumption versus the papers without a rise.

**Table 1:** The Change in Pornography Consumption in the United States of America and Canada Since 2000 Showing, in tabular form, the data that was extracted from the 21 papers used for part A [11-31]. Part A of our analysis is determining, via available previous research, if pornography consumption has increased in the United States and Canada since the year 2000.

Reference number	Year	Number of participants	Change in consumption	Synopsis
[11]	2015	1961	No effect	Pornography views increased due to the expanded availability and affordability of materials.
[12]	2020	1031	rise	Increased pornography consumption is linked to increased depression, anxiety, and stress.
[13]	2008	813	rise	Pornography use is positively associated with risky sexual behaviors, substance abuse patterns, and extramarital affairs.
[14]	2004	1792	decrease	Pornography consumption is associated to an increased risk of having partnered sexual relations.
[15]	2014	20,000	rise	Pornography use causes decreased coupled sexual intimacy, increased extramarital affairs and divorce rates.
[16]	2017	505	rise	Pornography use negatively impacted men partnered sexual relations and no change for women.
[17]	2012	4492	rise	Pornography views are related to decreased physical, sexual relations, increased isolation, and increased psychological disturbance.
[18]	2013	1215	rise	Pornography use is positively associated with neuroticism, compulsivity, emotional/psychological distress.
[19]	2013	365	no effect	Pornography consumption is associated with relationship alternatives and extra marital sexual relations.
[20]	2014	333	rise	Higher problematic pornography usage scores are positively associated with low self-esteem, poor mental health, decreased attachment & associated with other addictive disorders.
[21]	2013	221	rise	Pornography use demonstrates positive reinforcement similar to other addictive behaviors - pornography views correlate to increase masturbation with no change in partnered sexual relations.
[22]	2008	71	rise	Pornography users demonstrated more impulsive choice patterns.
[23]	2020	177	rise	Positive correlation between pornography users and depression, anxiety, and obsessive-compulsive behaviors.
[24]	2019	230	rise	Pornography exposure increase odds of casual sexual activity in unhappy people with no association in happy individuals.
[25]	2018	174	rise	Increase in arousal and desire for solo acts & associated with other addictive disorders.
[26]	2015	340	rise	Rise in porn use, negatively impacts daily function.
[27]	2018	1392	rise	Increased pornography views due to increase variety and accessibility - pornography use primarily for solo masturbation.
[28]	2020	9504	rise	Less partnered sexual activity with increased masturbation in both men and women coincides with increased pornography use.
[29]	2010	245	rise	Increase pornography consumption is related to exploring new sexual behaviors and relationships.
[30]	2012	1021	rise	Decrease in partnered sexual relations & increase in unhappiness.
[31]	2007	400	rise	Positive association between pornography users and loneliness.
		Total = 46,282		

Part B: COVID-19 and Its Effect on Pornography Consumption and Consequences on Sexual and Psychological Health

Next, we searched for articles on how the COVID-19 pandemic impacted pornography consumption. We then included the effect on sexual relationships; this includes how social isolation from the pandemic changed the dynamic of sexual encounters and how that impacted romantic and sexual relationships between people. After a review of all 42 papers was completed by all authors independently, 10 of those were used for part B of this paper. The total participants that demonstrated a rise in pornography viewing during the pandemic was N4 = 10,033. To get the total increase in pornography consumption during the pandemic we took the sum of N2 (42,164) and N4 (10,033) giving us a total sum of all pornography increases at N5 = 52,197. This analysis gave us the total number of participants for pornography consumption in parts A and B. To get the percentage of additional increase since COVID-19 compared to only the papers that had shown an increase in pornography consumption. We then took N4 divided by N5 multiplied by 100 to give us a 19% additional increase in pornography consumption since the COVID-19 pandemic. Data extracted from these papers are outlined in Table 2.

**Table 2:** Table 2: Impact of COVID-19 on Pornography Consumption and the Effect on Sexual Relations. Showing the data extracted for part B of our analysis, to determine if COVID-19 impacted the increase in pornography consumption, and if so, how that affected physical sexual relationships [32-40].

Reference number	Year	Number of participants	Synopsis
[32]	2021	247	Decrease overall sex drive, increase solo sex and depression during COVID19 pandemic.
[33]	2022	1171	No significant difference in pornography consumption before and during the COVID19 pandemic.
[34]	2022	1706	Increase addictive behaviors including pornography use during covid19.
[35]	2021	868	Covid 19 and social distancing caused initial increase in pornography views with problematic pornography use decreasing in men and remaining unchanged in women by August 2020.
[36]	2021	291	Increase depression, anxiety, alcohol use, and decrease relationship satisfaction during COVID19.
[37]	2020	1559	Increase solo and decrease partnered sex during COVID19 pandemic.
[38]	2020	2608	COVID19 and social isolation caused decrease in partnered sexual activity, increase in pornography use, masturbation, and incidence of depression and anxiety.
[39]	2021	351	Increase depression and anxiety during COVID19 with no changes in partnered sexual activity.
[40]	2021	1232	Increase pornography use during covid19 but no change in addictive porn use - depression and anxiety positively associated with pornography consumption.
		Total= 10,033	

Part C: Pornography Consumption and Its Relation to Other Addictive Disorders and Cluster B Personality Traits

Finally, we discuss if problematic pornography consumption is related to other addictive disorders and cluster B personality traits. Cluster B personality disorders include four groups: narcissistic, histrionic, antisocial, and borderline personality disorder. Cluster B personality disorders are characterized by overly dramatic and emotionally unpredictable thoughts and actions. The only DSM-5 addictive disorders are gambling, internet gaming disorder, alcohol use disorder, and substance use disorder. Although people can have compulsive behaviors of eating, shopping, and sex, just to name a few, these are not official diagnoses in the DSM-5. Addictive behaviors typically include compulsivity, impulsivity, neuroticism, and disinhibition to some degree. These addictive behaviors can accompany addictive disorders, cluster B personality disorders, and compulsive behaviors. We conducted separate searches for addictive disorders associated with pornography use and pornography use and its link to cluster B personality disorders. Of the 42 total articles, 10 of those were used for part C of this paper. Of the 57,173 total participants, only N6 = 16,771 participants fell into one of these categories. By taking the 16,771 participants that demonstrated addictive disorders or cluster B personality disorder traits and dividing that by the total number of participants, then multiplying that by 100, we could conclude that 36.6% of the studied population also exhibits addictive disorders or cluster B personality disorder traits. Shown below in Table 3 is how we took the data available to determine if there is any relationship between pornography consumption and other addictive behaviors [41-44]. This table also shows the relationship, or lack thereof, between pornography use and cluster B personality types [41,45-50].

**Table 3:** Pornography Consumption and Its Relation to Other Addictive Behaviors and Cluster B Personality Traits Showing data extracted from part C looking at the relationship between pornography consumption, other addictive behaviors and cluster B traits.

Reference number	Year	Number of Participants that fit into cluster B trait and/or addictive behaviors	Synopsis
[41]	2018	273	Pornography use is associated with antisocial personality disorder traits, alcohol use, drug use, and increased violent behaviors.
[42]	2018	13,778	Increased pornography use showed increased impulsivity and compulsivity.
[45]	2022	1,250	Impulsive and antisocial traits are associated with erotic content use.
[46]	2017	256	Narcissism was associated with extensive pornography consumption.
[47]	2019	142	Pornography view rates correspond to compulsivity, narcissistic and histrionic personality traits.
[48]	2014	257	Online pornography use positively corresponds to the level of narcissism.
[49]	2007	87	No correlation found between pornography consumption and borderline personality disorder.
[43]	2013	51	Pornography consumption is positively associated with shopping, spending, gambling, eating, and self-harm addictions.
[50]	2009	410	Pornography consumption is associated with antisocial behavior and disinhibition.
[44]	2018	267	Pornography consumption is associated with sex addiction and neuroticism.
		Total= 16,771	

Results 

Part A discusses the rise in pornography consumption since 2000 and how that has caused an increase in solo sexual acts and decreased partnered sexual encounters. All papers in this section of our analysis include participants who engage in solo sexual acts versus partnered sexual acts. Our results conclude that there has been a 91.10% total rise in pornography consumption and solo masturbation since 2000. This result suggests that individuals are becoming increasingly dependent on pornography as a source of sexual gratification, decreasing the frequency of sexual encounters with partners. The data also shows that for all the papers that fit the criteria for an increase in pornography consumption, there was a pooled portion of 0.911. A value of 0.0889 resulted from the pooled proportion of data which showed no increase in pornography consumption. The standard error (SE) for this data set is 0.464. The z score was then calculated using the formula [Z=(p1 - p2)/SE] where p1 is the pooled proportion rise (0.911), p2 is the pooled proportion no rise (0.089), and SE is the standard error (0.004*100). With a z score of 1.76 this data is supportive. Using a one-tailed test we get a p-value of 0.039 which is significant compared to p < 0.05. These results are outlined in Table 4. 

**Table 4:** The Total Rise in Pornography Consumption Showing the results for part A, the total rise in pornography consumption since 2000. The first column shows the total number of participants in the articles used. The second column shows the number of participants, from the total number of participants, that had increased pornography consumption. The third column shows the number of participants, from the total number of participants, that had a decrease or no change in pornography consumption. The last column shows the total rise.

Total Participants (N1)	Rise in Pornography Consumption Since 2000 (N2)	Decrease/No Change in Pornography Consumption Since 2000 (N3)	Total Participants/Total Decrease (No Change)*100 (N1/N3*100)
46,282	42,164	4,118	(42,164/46,282)*100
			Total rise: 91.10%

Part B uses the participants that demonstrated a rise in pornography consumption and solo masturbation from part A and compares them to the papers that depicted a rise in pornography consumption during the COVID-19 pandemic specifically. There was an additional 19% increase in pornography consumption due to the COVID-19 pandemic. These results are outlined in Table 5*.*

**Table 5:** Total Rise in Pornography Consumption During COVID-19 Showing the percentage of pornography consumption that increased further during the COVID-19 pandemic.

Rise in Pornography Consumption Since 2000 + Rise in Pornography Consumption During Pandemic (Sum N2+N4) N5	Rise in Pornography Consumption During COVID-19 (N4)	Rise in Pornography Consumption During COVID-19/Total Rise in Pornography Consumption*100 (N4/N5*100)
52,197	10,033	19%

Part C of our paper explored the associations between other addictive personality disorders and cluster B personality traits associated with pornography consumption. The total percentage of pornography viewers who also demonstrated other addictive disorders and/or cluster B personality traits yielded a pooled proportion for part C of 36.2%. Using the pooled proportion from part C we compared the results to the pooled proportion of part A to determine SE and z-score. SE between the two groups was 0.438 and the calculated z-score was 1.25. A two-tailed z-score of 1.25 corresponds to a p-value of 0.211 which is not significant compared to p < 0.05. This result is outlined in Table 6.

**Table 6:** Total Pornography Viewers With Other Associated Addictive Disorders or Cluster B Personality Traits Showing that there was not in fact any significant statistical evidence to support that pornography consumption is related to other addictive disorders and cluster B personality traits.

Total Participants (Nt)	Addictive Disorder/Cluster B Personality Trait (N6)	Addictive Disorders/Cluster B Personality Traits/Total Participants*100 (N6/Nt*100)
57,173	16,771	36.2 %

## Conclusions

The limitations of this paper include publication bias, lack of long-term follow-up post-COVID-19 pandemic, lack of a universal scoring system for problematic pornography use, potential recall biases from studies used, and compounding. Our presumption of pornography consumption increasing since the year 2000 was supported by the data gathered which yielded a p-value that was statistically significant. However, our theory that problematic pornography consumption was associated with other addictive disorders and cluster B personality traits was not supported, due to only 36.2% of participants who demonstrated an increase in pornography use since 2000 also demonstrated an additional addictive disorder or cluster B personality trait characteristics. This finding is not significant since it gave a p-value of 0.211 which is greater than a significance level of p < 0.05. We found that there has been an increase in pornography consumption since 2000 and an even further increase during the COVID-19 pandemic. We found that during this increase there has been an increase in solo masturbation and fewer partnered sexual acts, with an increase in depression, stress, and anxiety levels. We believe this is associated with pornography consumption but is also due to isolation during the pandemic since pornography use increased even more during that time. This is indicated by the population proportion calculated, showing a 91% increase in viewers who fell into the increased category. The z-score for this data was 1.76 which corresponds to a one-tailed p-value of 0.039 which is statistically significant compared to p < 0.05, indicating that the increase in consumption is supported by the data. The papers used in this meta-analysis are all from 2000 and more recent years to help associate pornography use with increased ease of accessibility. Since the 2000s the internet and social media have exploded. Because of this, erotic content has become more easily accessible and, at times, free of cost to consumers. The data collected from the papers for part B showed that the increase in pornography consumption during the pandemic was primarily driven by the increased time that individuals spent at home. With many people working from home and social distancing measures in place, individuals had more time on their hands. They turned to pornography as a source of entertainment and sexual gratification. 

With pornography becoming increasingly accessible, we theorize that problematic pornography will continue to rise, resulting in either a standardized way to quantify pornography use or utilizing the PPCS as the quantifier for a pornography addiction diagnosis. We believe that further research can be done on this topic to help solidify the association, or lack thereof, between other addictive disorders and cluster B personality traits with problematic pornography consumption. Continued research on this topic could aid in determining if pornography use has a place alongside other addictive disorder diagnoses in DSM-5.
